# Timing of Morphine Administration Differentially Alters Paraventricular Thalamic Neuron Activity

**DOI:** 10.1523/ENEURO.0377-19.2019

**Published:** 2019-12-17

**Authors:** Dillon S. McDevitt, Nicholas M. Graziane

**Affiliations:** 1 Departments of Anesthesiology and Perioperative Medicine and Pharmacology, Penn State College of Medicine, Hershey, PA 17033; 2 Neuroscience Graduate Program, Penn State College of Medicine, Hershey, PA 17033

**Keywords:** brain slice electrophysiology, circadian cycle, conditioned place preference, light/dark cycle, morphine, paraventricular thalamic nucleus

## Abstract

The paraventricular thalamic nucleus (PVT) is a brain region involved in regulating arousal, goal-oriented behaviors, and drug seeking, all key factors playing a role in substance use disorder. Given this, we investigated the temporal effects of administering morphine, an opioid with strongly addictive properties, on PVT neuronal function in mice using acute brain slices. Here, we show that morphine administration and electrophysiological recordings that occur during periods of animal inactivity (light cycle) elicit increases in PVT neuronal function during a 24-h abstinence time point. Furthermore, we show that morphine-induced increases in PVT neuronal activity at 24-h abstinence are occluded when morphine administration and recordings are performed during an animals’ active state (dark cycle). Based on our electrophysiological results combined with previous findings demonstrating that PVT neuronal activity regulates drug-seeking behaviors, we investigated whether timing morphine administration with periods of vigilance (dark cycle) would decrease drug-seeking behaviors in an animal model of substance use disorder. We found that context-induced morphine-seeking behaviors were intact regardless of the time morphine was administered (e.g., light cycle or dark cycle). Our electrophysiological results suggest that timing morphine with various states of arousal may impact the firing of PVT neurons during abstinence. Although, this may not impact context-induced drug-seeking behaviors.

## Abstract (precis)

The paraventricular thalamic nucleus (PVT) is a brain region involved in regulating arousal, goal-oriented behaviors, and drug seeking, all key factors playing a role in substance use disorder. Given this, we investigated the temporal effects of administering morphine, an opioid with strongly addictive properties, on PVT neuronal function in mice using acute brain slices.

## Significance Statement

Here, we show that the timing of morphine administration differentially alters paraventricular thalamic nucleus (PVT) neuronal function. Timing morphine administration during an animals’ inactive state increases PVT neuronal activity, while this morphine-induced effect is occluded when morphine administration occurs during an animals’ active state. Given evidence that PVT neuronal activity regulates drug-seeking behaviors, we investigated whether timing morphine administration with periods of vigilance would decrease drug-seeking behaviors in an animal model of substance use disorder. We found that morphine-induced conditioned place preference (CPP) was intact regardless of the time morphine was administered. Our results suggest that timing morphine with various states of arousal impacts the firing of PVT neurons during abstinence, but not morphine-induced CPP.

## Introduction

Opioids induce sleep-wake disturbances in humans and in rodents (Oyefeso et al., 1997; Stinus et al., 1998; Li et al., 2009), with evidence suggesting that sleep disturbances facilitate drug-seeking behaviors (Hasler et al., 2012; Logan et al., 2018). There has been substantial work dedicated to understanding the mechanisms mediating opioid-induced sleep disturbances, with a focus on opioid-induced changes in genes and proteins whose expression is driven by circadian cycles [e.g., circadian locomotor output cycles kaput (CLOCK) or period (Per1, Per2 and Per3); Hasler et al., 2012; Logan et al., 2014]. However, brain regions that link opioid-induced sleep disturbances with drug-seeking behaviors remains largely unknown.

The paraventricular nucleus of the thalamus (PVT) is a midline thalamic nucleus that receives dense innervation from brain regions, including the locus coeruleus, dorsal raphe, penduculopontine tegmental nucleus, orexin neurons in the hypothalamus, and suprachiasmatic nucleus, that are involved in regulating wakefulness (the presence of voluntary motor activation and responsiveness to internal and external stimuli), and rapid eye movement (REM), and non-REM (NREM) sleep patterns (Novak et al., 2000; Peng and Bentivoglio, 2004; Li and Kirouac, 2012; Kirouac, 2015; Scammell et al., 2017). Additionally, the PVT projects to several regions of the greater reward circuit, including the nucleus accumbens, amygdala, and medial prefrontal cortex (Li and Kirouac, 2008; Kirouac, 2015; Dong et al., 2017). Functionally, the PVT regulates wakefulness (Herrera et al., 2016; Mátyás et al., 2018; Ren et al., 2018), with arousal/awake states coinciding with increases in PVT neuronal activity (Kolaj et al., 2012). In parallel, cocaine, a drug of abuse that disrupts sleep cycles (Schierenbeck et al., 2008) and promotes drug-seeking (Liu et al., 2016), increases PVT neuronal excitability (Yeoh et al., 2014), while reducing PVT activity suppresses cocaine or alcohol-seeking behaviors (Hamlin et al., 2009; James et al., 2010; Browning et al., 2014; Neumann et al., 2016). Given this, we investigated the effects of morphine, an opioid with high risk of addiction and known to induce sleep disturbances (Kay, 1975; Shaw et al., 2005; Dimsdale et al., 2007; Robertson et al., 2016), on PVT neuronal function. To test this, we used whole-cell patch clamp electrophysiology in mouse brain slices to investigate morphine-induced intrinsic and synaptic effects on PVT neurons 24 h following repeated morphine administration. Additionally, we investigated whether morphine-induced alterations on PVT neuronal function may be differentially affected by the timing of morphine administration, which we tested by administering morphine during animals’ inactive (light cycle) or active states (dark cycle) when PVT neuronal function is decreased or increased, respectively. Finally, given the evidence that the PVT is influenced by circadian rhythms (Peng et al., 1995; Novak and Nunez, 1998; Kolaj et al., 2012), we investigated whether timing morphine administration with PVT activity states impacted morphine-induced conditioned place preference (CPP), a model of addiction-like behavior (Bardo and Bevins, 2000) that is influenced by the circadian cycle (Abarca et al., 2002; Kurtuncu et al., 2004).

## Materials and Methods

### Animals

**Figure 1.**
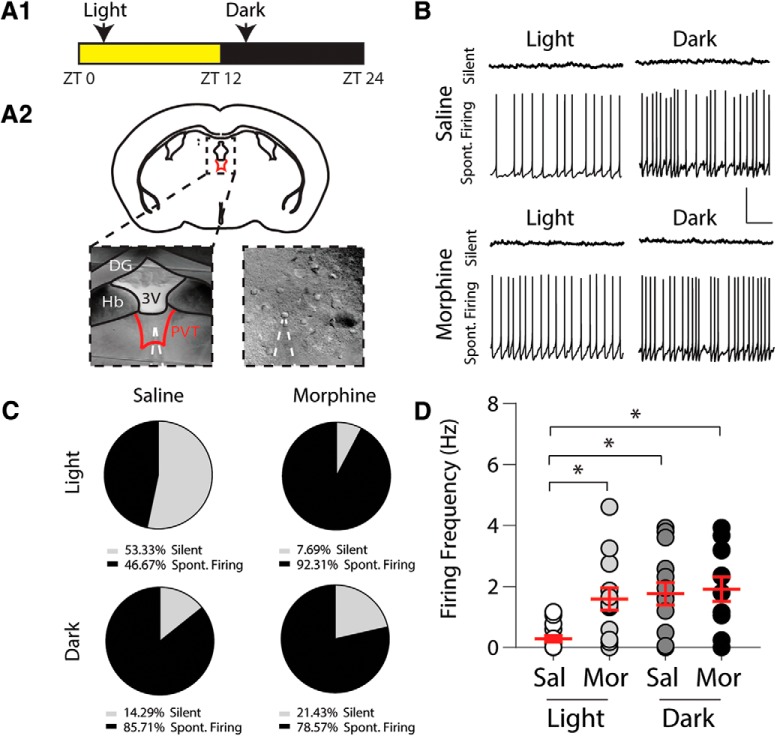
Morphine administration during the light cycle increases the number of spontaneously firing PVT neurons. ***A1***, ZT timeline showing times in which acute brain slices were prepared. Slices were prepared at either ZT2 (light) or ZT14 (dark). ***A2***, Coronal mouse brain slice showing an electrophysiological recording in the PVT (dashed shape outlines the recording electrode that is patched onto a PVT neuron). ***B***, Representative traces showing the firing of PVT neurons 24 h following saline or morphine administration during light or dark cycles. ***C***, Quantification of silent or spontaneously firing PVT neurons following repeated saline or morphine treatment. ***D***, Summary showing the overall firing frequency of PVT neurons 24 h following repeated saline (Sal) or morphine (Mor) administration (*F*_(3,52)_ = 5.52, *p* = 0.002, one-way ANOVA); *p < 0.05. Scale bars: 40 mV, 2 s.

All experiments were done in accordance with procedures approved by the Pennsylvania State University College of Medicine Institutional Animal Care and Use Committee Institutional Animal Care and Use Committee. Male and female C57BL/6 mice aged 5–10 weeks were purchased from The Jackson Laboratory (stock #000664), singly housed, and maintained on a regular 12/12 h light/dark cycle (lights on 7 A.M., lights off 7 P.M.) with *ad libitum* food and water. For dark cycle electrophysiological experiments (Figs. 1, 3, and 4), mice were switched to a lights on 7 P.M., lights off 7 A.M. schedule and allowed to adapt to the new cycle for two weeks before receiving injections. We found that two weeks of acclimation is sufficient to promote diurnal variations in PVT neuronal firing patterns in mice (Fig. 1) as previously observed in rats (Kolaj et al., 2012). For dark cycle CPP experiments, animals were housed on a regular 12-h light/dark cycle. Since no significant differences were observed between sexes in our electrophysiological experiments (Table 1), all behavioral tests were run with male C57BL/6 mice aged 5–10 weeks (The Jackson Laboratory, stock #000664) who were singly housed.

**Table 1.** Sex comparisons within electrophysiological assessments

	Saline			Morphine		
Experiment	Male	Female	*p* value	Male	Female	*p* value
Spontaneously firing light (Fig. 1)	0.34 ± 0.22 (5)	0.27 ± 0.13 (10)	0.91	1.41 ± 1.07 (4)	1.67 ± 0.31 (9)	0.75
Spontaneously firing dark (Fig. 1)	1.77 ± 0.24 (4)	1.62 ± 0.51 (10)	0.85	2.22 ± 0.59 (3)	1.83 ± 0.50 (11)	0.71
IME light 100 pA (Fig. 2)	7.88 ± 1.67 (8)	9.92 ± 2.11 (13)	0.50	16.87 ± 1.86 (15)	11.00 ± 4.71 (4)	0.19
IME dark 100 pA (Fig. 3)	25.2 ± 4.76 (5)	20.35 ± 1.33 (14)	0.19	17.28 ± 2.59 (7)	21.00 ± 0.82 (14)	0.10
AMPA/NMDA ratios light (Fig. 4)	0.77 ± 0.05 (4)	0.85 ± 0.11 (5)	0.55	1.46 ± 0.41 (3)	1.27 ± 0.12 (5)	0.59
AMPA/NMDA ratios dark (Fig. 4)	1.43 ± 0.23 (5)	1.05 ± 0.08 (6)	0.14	1.01 ± 0.26 (3)	1.19 ± 0.14 (8)	0.75

Mean ± SEM; number of cells (*n*); Student’s *t*-test was used for statistical measures.

### Drugs

(−)-Morphine sulfate pentahydrate was provided by the National Institute on Drug Abuse Drug Supply Program. NBQX and AP5 were purchased from Tocris Biosciences.

### Repeated systemic injections of saline or morphine

Before drug administration, mice were allowed to acclimate to their home cages for >5 d. For drug treatment, we used a 5-d repeated drug administration procedure (Graziane et al., 2016). In all electrophysiological experiments (Figs. 1-Figs. 4), once per day for 5 d, mice were taken out of the home cages at Zeitgeber time (ZT)2 (for experiments performed during the light cycle) or ZT14 (for experiments performed during the dark cycle; ZT0 = lights on, ZT12 = lights off) for an intraperitoneal injection of either (−)-morphine sulfate pentahydrate (10 mg/kg in saline) or the same volume of saline, and then placed back to the home cage. Animals were randomly selected for each drug treatment. Morphine-treated or saline-treated animals were then used for electrophysiological recordings ∼24 h following the last injection. As previously published (Robinson and Kolb, 1999), this drug-treatment paradigm does not produce noticeable signs of withdrawal and was chosen because it induces locomotor sensitization and CPP (Spanagel et al., 1998; Mueller et al., 2002; Graziane et al., 2016).

### Acute brain slice preparation

Mice were deeply anesthetized with isoflurane and cardiac perfused with an ice-cold NMDG-based cutting solution containing the following: 135 mM *N*-methyl-d-glucamine, 1 mM KCl, 1.2 mM KH_2_PO_4_, 0.5 mM CaCl_2_, 1.5 mM MgCl_2_, 20 mM choline-HCO_3_, and 11 mM glucose, saturated with 95%O_2_/5%CO_2_, adjusted to pH 7.4 with HCl, osmolality adjusted to 305. Following perfusion, mice were decapitated and brains were rapidly removed; 250-μm coronal brain slices containing the PVT were prepared, via a Leica VT1200s vibratome, in 4°C NMDG cutting solution, and the lateral hemispheres (∼2.5 mm lateral from the midline) were removed to allow for the slices to fit into the recording chamber. Before recording, slices were allowed to recover in artificial cerebral spinal fluid (aCSF) containing the following: 119 mM NaCl, 2.5 mM KCl, 2.5 mM CaCl_2_, 1.3 mM MgCl_2_, 1 mM NaH_2_PO_4_, 26.2 mM NaHCO_3_, and 11 mM glucose, osmolality of 290, at 31°C for 30 min followed by 30 min at 20–22°C. After a 1-h recovery period, slices were kept at 20–22°C for the rest of the recording day.

### Electrophysiology

Whole-cell recording. All recordings were made from the PVT of mice spanning between bregma –0.94 and –2.18 mm (Paxinos and Franklin, 2004). Therefore, we randomly sampled neurons located in the middle PVT (Paxinos and Franklin, 2004) corresponding to a region that projects to the reward neurocircuit (Li and Kirouac, 2008; Zhu et al., 2016; Li et al., 2018) and is implicated in wakefulness (Ren et al., 2018). These coordinates exclude what are considered the anterior and posterior PVT (Paxinos and Franklin, 2004). Recordings within bregma –0.94 and –2.18 mm were evenly sampled with no bias between experimental groups. Slices were transferred to a recording chamber and neurons were visualized using infrared differential interference contrast microscopy. During recording, slices were superfused with aCSF at room temperature. For recordings of spontaneously firing neurons (Fig. 1), recording electrodes [2–5 MΩ; borosilicate glass capillaries (WPI #1B150F-4) pulled on a horizontal puller from Sutter Instruments (model P-97)] were filled with a potassium-based internal solution containing the following: 130 mM KMeSO_3_, 10 mM KCl, 10 mM HEPES, 0.4 mM EGTA, 2 mM MgCl_2_-6H_2_0, 3 mM Mg-ATP, and 0.5 mM Na-GTP, pH 7.2–7.4. Immediately following whole-cell configuration, spontaneous activity was analyzed over a 100 s duration. As previously shown, cell dialysis of the internal solution in whole-cell patch-clamp configuration does not impact the spontaneous activity of PVT neurons (Kolaj et al., 2012). For intrinsic membrane excitability experiments, recording electrodes (2–5 MΩ) were filled with a potassium-based internal solution (see above in the Electrophysiology subsection of the Materials and Methods). Resting membrane potential was recorded immediately following break-in. Before beginning the protocol, cells were adjusted to a resting membrane voltage of –60 mV. This typically was achieved with <30 pA current injection, and cells were discarded if the current needed to adjust the cell to –60 mV was >50 pA. A current step protocol, consisting of 600 ms steps ranging from –100 to +100 pA in 20 pA increments, was conducted with a 20 s intrasweep interval. The number of action potentials observed at each current step was recorded. I_H_ currents were calculated by measuring the amplitude of the peak current minus the steady-state current at a –60 pA hyperpolarizing step. Cells were classified into a firing phenotype based off of their firing behavior at the +100 pA step. We observed four major phenotypes: tonic firing, initial burst firing, delayed burst firing, and initial single spike. For AMPA receptor (AMPAR)/NMDA receptor (NMDAR) ratio experiments, recording electrodes (2–5 MΩ) were filled with a cesium-based internal solution the following: 135 mM CsMeSO_3_, 5 mM CsCl, 5 mM TEA-Cl, 0.4 mM EGTA (Cs), 20 mM HEPES, 2.5 mM Mg-ATP, 0.25 mM Na-GTP, and 1 mM QX-314 (Br), pH 7.2–7.4. To isolate excitatory currents, picrotoxin (100 μM) was included in the aCSF. To evoke postsynaptic currents, presynaptic afferents were stimulated via a constant-current stimulator (Digitimer) using a monopolar stimulating electrode (glass pipette filled with aCSF) positioned 100 μm away from and along the same *z*-axis as the recorded neuron. Cells were held at +40 mV for the duration of the experiment. Once a stable baseline was observed, 50 traces were recorded. Following this, NBQX (2 μM) was bath applied to isolate NMDAR-mediated currents. The drug was allowed to wash on, and 50 more sweeps were recorded. The AMPAR-mediated current was then obtained via digital subtraction of the NMDAR-mediated current from the mixed current. The AMPAR/NMDAR ratio was then calculated by taking the peak amplitude of the AMPAR-mediated current divided by the peak amplitude of the NMDAR-mediated current. Currents were recorded with either an Axon Multiclamp 700B amplifier or Sutter Double IPA, filtered at 2–3 kHz, and digitized at 20 kHz. For all recordings, series resistance was typically 10–25 MΩ, left uncompensated, and monitored throughout. Cells with a series resistance variation >20% were discarded from analysis.

### CPP

CPP chambers (Med Associates), located in the mouse housing room, consisted of three distinct compartments separated by manual guillotine-style doors. Each compartment had distinct contextual characteristics: the middle (neutral) compartment (2.85″ × 5″ × 5″) had gray walls and gray plastic floor, while the choice compartments (6.6″ × 5″ × 5″ each) had either white walls and stainless-steel mesh floor or black walls and stainless-steel grid floor. All compartments were illuminated with a dim light during use. Immediately following use the entire preference chamber was cleaned thoroughly with a scent-free soap solution. Mouse locations, activity counts, and time spent in each compartment were collected via automated data-collection software (Med Associates) via infrared photobeam strips lining each compartment.

#### Habituation

During light or dark cycles, mice were placed in the center compartment with free access to all three compartments for 20 min once a day for 2 d. Time spent (seconds) in each compartment was recorded.

#### Conditioning

Twenty-four hours after habituation, mice received 5 d of conditioning training. Morphine-paired compartments were assigned based on the least preferred side (a biased approach; Tzschentke, 2007), calculated by averaging time spent in each compartment over the two habituation days. Similar to conditioning studies with alcohol (Gremel et al., 2006), we find that C57BL/6 mice will reliably develop morphine CPP using a biased approach. During conditioning, mice received an injection of saline and were placed into the most preferred compartment for 40 min; 6 h later, mice received an injection of saline (control group) or morphine (10 mg/kg, i.p.) and were placed into their least preferred compartment for 40 min (Koo et al., 2014).

#### Postconditioning

Twenty-four hours after the last conditioning day, mice were placed in the center compartment, where they were allowed to move freely for 20 min. Our postconditioning took place at a time point corresponding to 3 h before drug conditioning (e.g., morphine conditioning took place at ZT8, postconditioning took place the next day at ZT5). CPP scores were calculated as time spent in the drug-paired side minus the time spent on the same side during the preconditioning day (Bohn et al., 2003). Activity counts are defined as any beam break within a current zone. This is inclusive of grooming, rearing, and lateral movements.

### Statistical analysis

All results are shown as mean ± SEM. Each experiment was replicated in at least three animals. No data points were excluded. Sample size was presented as n/m, where “n” refers to the number of cells and “m” refers to the number of animals. Statistical significance was assessed in GraphPad Prism software using χ^2^ analysis, a one-way or two-way ANOVA with Bonferroni’s correction for multiple comparisons as specified. F values for two-way ANOVA statistical comparisons represent interactions between variables unless otherwise stated. Our goal, a priori, was to examine pairwise comparisons between drug treatment and time of drug treatment regardless if the interaction effect between drug treatment and cell type was strong. Thus, before analysis, we created all possible independent groups based on drug treatment and cell type combinations and performed a one-way ANOVA with pairwise comparisons. The results from these pairwise comparisons from this one-way ANOVA would be equivalent to performing a two-way ANOVA with an interaction term (drug treatment, cell type, drug treatment × cell type interaction) and then performing *post hoc* pairwise comparisons on the interaction term from the two-way ANOVA model. Two-tail tests were performed for all studies.

## Results

### Morphine increases basal PVT neuronal firing

Evidence suggests that PVT neurons in nocturnal rats display diurnal variations in basal firing patterns such that increases in PVT neuronal activity are associated with increases in activity/arousal states (Kolaj et al., 2012). Similarly, in nocturnal mice expressing increases in activity/arousal during the dark cycle (Schwartz and Zimmerman, 1990), we observed a greater number of spontaneously firing PVT neurons when electrophysiological recordings took place during saline-treated animals’ dark cycle (χ^2^(1) = 4.89, *p* = 0.03, χ^2^ analysis; Figs. 1*B*–*D*).

We next tested the effects that repeated morphine (10 mg/kg, i.p.) exposure had on PVT neuron spontaneous firing and found that 24 h following a 5 d, once daily repeated morphine injection paradigm during the light cycle, there was an increase in the number of spontaneously firing PVT neurons compared to saline-treated control mice (χ^2^(1) = 6.65, *p* = 0.01, χ^2^ analysis; Fig. 1*C*), likely stemming from the depolarized resting membrane potential in PVT neurons from morphine-treated mice (Table 2; Kolaj et al., 2012). In contrast, the morphine-induced increase in spontaneously firing PVT neurons was likely occluded during dark cycle administration as saline-treated control animals showed increases in spontaneous neuronal firing (χ^2^(1) = 0.244, *p* = 0.62, χ^2^ analysis; Fig. 1*C*). Furthermore, a one-way ANOVA *post hoc* analysis revealed that the overall firing frequency was significantly increased in morphine-treated animals during the light cycle (Bonferroni *post hoc* test, *p* = 0.041), while during the dark cycle, this morphine-induced increase was likely occluded (Bonferroni *post hoc* test, light saline vs dark saline: *p* = 0.012; dark saline versus dark morphine: *p* > 0.999; Fig. 1*D*).

**Table 2.** Intrinsic properties of PVT neurons following saline or morphine treatment during the light or dark cycle

	Saline light	Morphine light	Saline dark	Morphine dark
RMP (mV)	–59.36 ± 0.90 (33)	–54.89 ± 0.95 (32)**	–51.38 ± 1.04 (33)**^	–50.77 ± 0.64 (36)**^^
Capacitance (pF)	56.57 ± 3.61 (23)	56.79 ± 3.48 (19)	64.27 ± 4.49 (22)	62.58 ± 4.25 (24)
Membrane resistance (MΩ)	562.4 ± 54.2 (23)	563.0 ± 75.79 (19)	1149 ± 129 (22)**^^	1193 ± 166.2 (24)**^^
Voltage sag (mV)	4.09 ± 0.40 (15)	4.85 ± 1.08 (15)	6.69 ± 1.17 (16)*	6.23 ± 1.82 (19)

Passive properties were compared across treatment groups using independent samples *t* tests. Mean ± SEM; number of cells (*n*); *significantly different from saline light; ^signifcantly different from morphine light; * or ^*p* < 0.05, ** or ^^*p* < 0.01. RMP, resting membrane potential.

### Increased PVT intrinsic neuronal excitability following morphine is dependent on the timing of morphine administration

**Figure 2.**
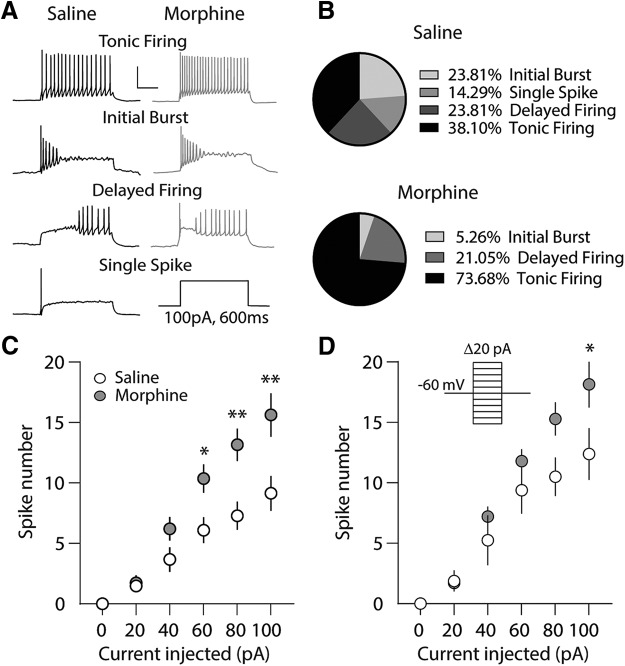
Repeated morphine exposure during the light cycle increases intrinsic membrane excitability of PVT neurons recorded in the light cycle. ***A***, Example traces demonstrating the four firing phenotypes observed in response to a depolarizing current injection (100 pA for 600 ms); tonic firing, initial burst, delayed firing, and single spike (saline, left; morphine, right). Single spike firing patterns were not observed in neurons from animals treated with repeated morphine. Scale bars: 40 mV, 200 ms. ***B***, Quantification of firing phenotypes in PVT neurons following repeated saline or morphine treatment. ***C***, Summary showing that 24 h following repeated morphine injections, the number of action potentials fired in PVT neurons is significantly increased at current injections of 60, 80, and 100 pA (saline: *n* = 21/5; morphine: 19/6; *F*_(5,190)_ = 5.14, *p* = 0.0002; two-way repeated measures ANOVA with Bonferroni *post hoc* test). ***D***, Summary showing that the number of action potentials fired from PVT neurons expressing tonic firing patterns 24 h after the last saline or morphine injection was significantly different at 100-pA current injection (saline: *n* = 8/3; morphine: 14/4; *F*_(5,100)_ = 2.41, *p* = 0.042; two-way repeated measures ANOVA with Bonferroni *post hoc* test); *p < 0.05, **p < 0.01.

In the PVT, changes in basal firing frequency are associated with changes in intrinsic membrane excitability (Kolaj et al., 2012), which sets the action potential threshold and determines, in part, the firing frequency (Daoudal and Debanne, 2003; Huang et al., 2011). We investigated the effects that repeated morphine administration has on the intrinsic membrane excitability of PVT neurons and tested whether these potential changes are influenced by the animals’ activity states. Using whole-cell electrophysiological recordings, we measured the number of action potentials in response to depolarizing currents, as this approach is often used to measure intrinsic membrane excitability (Desai et al., 1999; Nelson et al., 2003; Zhang and Linden, 2003; Ishikawa et al., 2009; Wang et al., 2018). In mice receiving saline injections during the light cycle, we observed four firing phenotypes 24 h following the last saline injection; initial burst, single spike, delayed firing, and tonic firing (Fig. 2*A*). The distribution of recorded neurons after saline treatment (*n* = 21 cells, five animals) was 38.10% tonic, 23.81% initial burst, 23.81% delayed, and 14.29% single-spike (Fig. 2*B*). Twenty-four hours following morphine administration during the light cycle, the intrinsic membrane excitability of PVT neurons (*n* = 19, six animals) displayed only three firing phenotypes (tonic, initial burst, and delayed firing), lacking only single spikes (distribution: 73.68% tonic, 5.26% initial burst, and 21.05% delayed firing neurons; Fig. 2*B*). A χ^2^ analysis revealed no significant interaction between morphine exposure (i.e., saline vs morphine treatment) and firing type during the light cycle (χ^2^(3) = 7.33, *p* = 0.06). However, we found that PVT neurons from morphine-treated animals had a significant increase in membrane excitability compared to saline controls during light cycle administration at current injections of 60, 80, and 100 pA (Bonferroni *post hoc* test, 60 pA: *p* = 0.029; 80 pA: *p* = 0.0007; 100 pA: *p* = 0.0001; Fig. 2*C*). To test whether the observed morphine-induced increase in PVT neuronal membrane excitability was due to increases in the proportion of tonically firing neurons or a selective increase in action potential frequency of tonically firing neurons, we repeated our analysis by focusing on only neurons that tonically fired (Fig. 2*D*). In doing so, we found that morphine significantly increased the number of action potentials at the maximum current injected (Bonferroni *post hoc* test, 100 pA: *p* = 0.020). These results suggest that the observed significant increases in intrinsic membrane excitability at submaximal current injections (i.e., 60–80 pA) were attributed to a shift in the proportion of neurons exhibiting greater evoked firing frequencies, while at higher current injections (i.e., 100 pA), this shift was attributed to increases in the proportion of neurons exhibiting greater evoked firing frequencies and/or due to increases in action potential spike number in tonically firing neurons.

**Figure 3.**
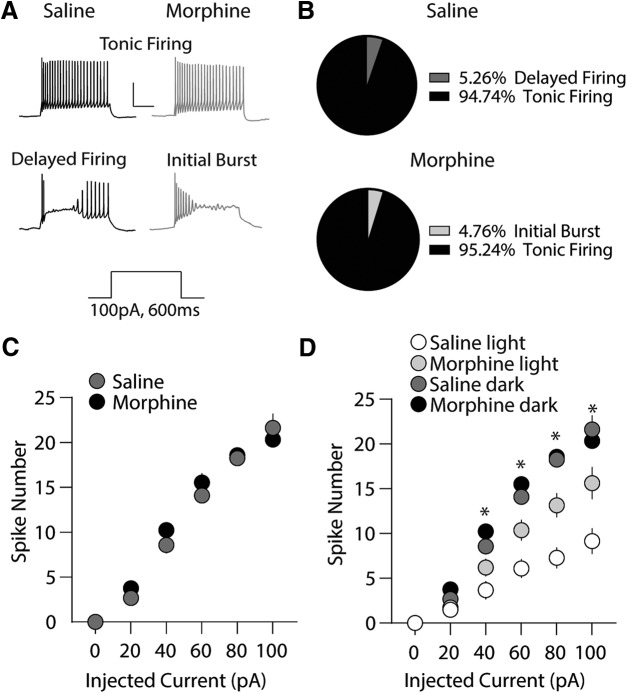
Repeated morphine exposure during the dark cycle has no effect on intrinsic membrane excitability of PVT neurons recorded in the dark cycle. ***A***, Example traces demonstrating the two firing phenotypes observed in response to a depolarizing current injection (100 pA for 600 ms); tonic and delayed firing (saline, left; morphine, right). PVT neurons from morphine-treated animals only expressed tonic firing responses to current injected. Scale bars: 40 mV, 200 ms. ***B***, Quantification of firing phenotypes in PVT neurons following repeated saline or morphine treatment. ***C***, Summary showing that 24 h following repeated morphine injections during the dark cycle, the number of action potentials fired in PVT neurons is unaltered (*F*_(5,190)_ = 1.41, *p* = 0.221; two-way repeated measures ANOVA with Bonferroni *post hoc* test; saline: *n* = 19/5; morphine: 21/6). ***D***, Summary showing intrinsic membrane excitability of PVT neurons recorded during the light or dark cycle in saline-treated or morphine-treated animals (Bonferroni *post hoc* test; 40 pA: saline light versus saline dark**, morphine light vs morphine dark**, saline light vs morphine dark*; 60 pA: saline light versus saline dark**, morphine light vs morphine dark**, saline light vs morphine dark**, morphine light vs saline dark*, saline light vs morphine light**; 80 pA: saline light vs saline dark**, morphine light vs morphine dark**, saline light vs morphine dark**, morphine light vs saline dark**, saline light vs morphine light**; 100 pA: saline light vs saline dark**, morphine light vs morphine dark**, saline light vs morphine dark**, morphine light vs saline dark**, saline light vs morphine light**; *p < 0.05, **p < 0.01; light = light cycle; dark = dark cycle.

We next investigated whether morphine-induced increases in PVT neuronal intrinsic membrane excitability were maintained following morphine injections and subsequent recordings during the animals’ active state (i.e., dark cycle). Our results show that PVT neurons (*n* = 19 cells, 5 animals) recorded from animals treated with saline during the dark cycle exhibited two firing phenotypes; tonic firing (94.74%) or delayed-firing (5.26%). A χ^2^ analysis revealed a significant interaction between the timing of saline exposure (i.e., saline-treated during the light vs dark cycle) and firing type (χ^2^(3) = 14.45, *p* = 0.002), which is consistent with previous findings (Kolaj et al., 2012). In animals treated with morphine during their dark cycle, PVT neurons (*n* = 21 cells, six animals) exhibited tonic firing (Fig. 3*A*,*B*) and initial burst, but lacked delayed firing, which was observed when morphine was administered during the animals’ light cycle (Fig. 2*B*). Despite the difference in neuronal firing patterns in morphine-light cycle versus morphine-dark cycle groups, a χ^2^ analysis revealed no significant interaction between the timing of morphine exposure and firing type (χ^2^(2) = 4.97, *p* = 0.08). Furthermore, an analysis of PVT neuronal intrinsic membrane excitability in dark cycle saline-treated versus morphine-treated animals revealed no significant differences (*F*_(5,190)_ = 1.41, *p* = 0.221; two-way repeated measures ANOVA; Fig. 3*C*) suggesting that PVT neurons are maximally excited during periods of activity, thus occluding morphine-induced alterations in PVT excitability. This interpretation is supported by no significant interactions between morphine exposure and firing type during the dark cycle (χ^2^(2) = 2.01, *p* = 0.38).

We next compared PVT neuronal membrane excitability in animals treated with saline during the dark cycle with animals treated with morphine during the light cycle. This comparison was made to investigate whether morphine-induced increases in PVT neuronal membrane excitability mimic the PVT neuronal membrane excitability that occurs during the active, dark cycle of saline-treated animals. The results show that dark cycle saline-treated animals express significantly greater evoked action potential firing versus light cycle morphine-treated animals (*F*_(15,380)_ = 9.86, *p* < 0.0001; two-way repeated measures ANOVA; Fig. 3*D*). These results suggest that administration of morphine during periods of inactivity (i.e., light cycle) are sufficient to increase PVT neuron intrinsic membrane excitability, but not to levels comparable to those observed during the animals’ active state.

### Morphine exposure during the light cycle increases AMPAR/NMDAR ratios on PVT neurons

Given that excitatory ionotropic glutamate receptor activation is a key regulator of PVT neuronal action potential firing (Hermes and Renaud, 2011), we investigated whether excitatory synaptic transmission was altered on PVT neurons following morphine exposure and whether any potential changes were dependent on the timing of morphine administration. To assess potential changes in excitatory postsynaptic strength, we measured AMPAR/NMDAR ratios in saline or morphine-treated animals in both the light and dark cycles. AMPAR/NMDAR ratio is relatively independent of the number of synapses, presynaptic release probability, and other presynaptic factors (Graziane and Dong, 2016). Therefore, a change in this ratio should reflect changes in postsynaptic AMPARs or NMDARs.

**Figure 4.**
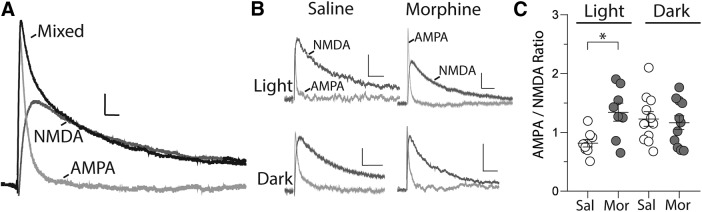
Twenty-four hours following repeated morphine injections during the light cycle, AMPAR/NMDAR ratios are increased on PVT neurons. ***A***, Example traces showing pharmacological separation of AMPAR and NMDAR EPSCs. Scale bars: 12.5 pA, 10 ms. ***B***, Example traces illustrating AMPAR-mediated and NMDAR-mediated currents recorded from neurons in the PVT 24 h following the last saline or morphine injection. Scale bars: 12.5 pA, 50 ms. ***C***, Summary showing a significantly increased AMPAR/NMDAR ratio in PVT neurons following repeated morphine injection during the light cycle but not during the dark cycle (*F*_(3,35)_ = 3.30, *p* = 0.032, one-way ANOVA with Bonferroni *post hoc* test; *n* = cells/mice: light cycle: saline (Sal) = 9/5, morphine (Mor) = 8/4; dark cycle: saline = 11/6, morphine = 11/6); *p < 0.05; light = light cycle; dark = dark cycle.

Using whole-cell recordings, we observed a significant increase in the AMPAR/NMDAR ratios in morphine-treated animals relative to saline controls when treatments or recordings were performed during the light cycle (one-way ANOVA with Bonferroni *post hoc* test, *p* = 0.036; Fig. 4*A–C*). In contrast, no significant difference in AMPAR/NMDAR ratios were observed between saline or morphine-treated animals when treatments or recordings took place during the dark cycle (one-way ANOVA with Bonferroni *post hoc* test, *p* > 0.999; Fig. 4*C*). These results highlight that morphine-induced alterations in glutamatergic transmission on PVT neurons is dependent on the timing of morphine administration.

### Morphine-induced place preference is not modulated by the timing of morphine administration

**Figure 5.**
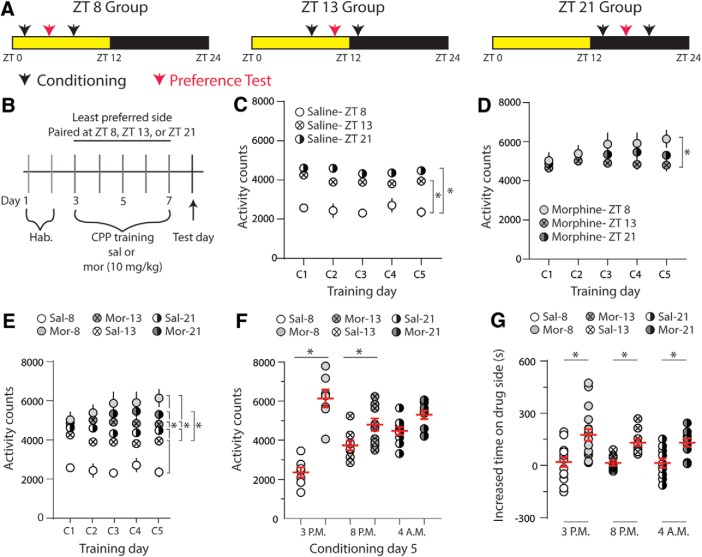
The time of day of morphine conditioning does not influence morphine-induced CPP. ***A***, CPP experimental groups. ***B***, Time line of CPP procedure. Mice were allowed to acclimate to the chambers for 2 d during habituation (Hab). During conditioning (days 3–7), mice were injected with saline (sal) or morphine (mor; 10 mg/kg, i.p.) and paired with the least preferred side at either ZT8, ZT13, or ZT21 CPP tests were performed 24 h postconditioning. ***C***, Summary showing activity counts for saline-treated animals during conditioning days (C1–C5) at each time point measured (ZT8, ZT13, or ZT21) [saline (Sal)-ZT8: *n* = 7; saline (Sal)-ZT13: *n* = 8; saline (Sal)-ZT21: *n* = 10; *F*_(8,88)_ = 0.766, *p* = 0.63; two-way repeated measures ANOVA with Bonferroni *post hoc* test]; *p < 0.05. ***D***, Summary showing activity counts for morphine-treated animals during conditioning days (C1–C5) at each time point measured (ZT8, ZT13, or ZT21; morphine (Mor)-ZT8: *n* = 7; morphine (Mor)-ZT13: *n* = 10; morphine (Mor)-ZT21: *n* = 10; *F*_(8,96)_ = 1.48, *p* = 0.17; two-way repeated measures ANOVA with Bonferroni *post hoc* test]; *p < 0.05. ***E***, Summary showing activity counts for saline (Sal)-treated or morphine (Mor)-treated animals during conditioning days (C1–C5) at each time point measured (ZT8, ZT13, or ZT21); [saline (Sal)-ZT8: *n* = 7; morphine (Mor)-ZT8: *n* = 7; saline (Sal)-ZT13: *n* = 8; morphine (Mor)-ZT13: *n* = 10; saline (Sal)-ZT21: *n* = 10; morphine (Mor)-ZT21: *n* = 10; *F*_(20,184)_ = 2.08, *p* = 0.006, two-way repeated measures ANOVA with Bonferroni *post hoc* test]; *p < 0.05. ***F***, Summary showing activity counts for saline (Sal)-treated or morphine (Mor)-treated animals mice on conditioning day 5 at each time point measured [saline (Sal)-ZT8: *n* = 7; morphine (Mor)-ZT8: *n* = 7; saline (Sal)-ZT13: *n* = 8; morphine (Mor)-ZT13: *n* = 10; saline (Sal)-ZT21: *n* = 10; morphine (Mor)-ZT21: *n* = 10; *F*_(5,46)_ = 17.6, *p* < 0.0001, one-way ANOVA with Bonferroni *post hoc* test]; *p < 0.05. ***G***, Summary showing that the time of day of morphine conditioning does not influence morphine-induced CPP [saline (Sal)-ZT8: *n* = 14; morphine (Mor)-ZT8: *n* = 17; saline (Sal)-ZT13: *n* = 8; morphine (Mor)-ZT13: *n* = 10; saline (Sal)-ZT21: *n* = 10; morphine (Mor)-ZT21: *n* = 10; *F*_(2,63)_ = 0.267, *p* = 0.767; two-way ANOVA with Bonferroni *post hoc* test]; *p < 0.05.

Given that (1) PVT activity plays a role in drug-seeking behaviors (Hamlin et al., 2009; Browning et al., 2014; Haight et al., 2015) and (2) our results show that morphine differentially influences PVT activity based on the timing of morphine treatment, we investigated whether the timing of morphine injections impacts addiction-like behaviors using the CPP model (Napier et al., 2013). Mice received daily, alternating conditioning for 40 min either with saline or with drug (saline control or morphine), separated by 6 h for 5 d (Fig. 5*A*,*B*), which has previously been shown to produce robust morphine-induced CPP (Graziane et al., 2016). Morphine (10 mg/kg, i.p.) pairings (or saline control) were administered at ZT8, ZT13, or ZT21 [saline pairings in the most preferred compartment took place 6 h prior; 12/12 h light/dark cycle; lights on ZT0 (7 A.M.) to ZT12 (7 P.M.)]. The time points of ZT8 and ZT13 are in accordance with our electrophysiological assessments during the light and dark cycles, respectively (Figs. 1A and 5A). The ZT21 time point was selected because it allowed us to control for potential confounding factors caused by light/dark cycle-induced variations in conditioning, i.e., animals were conditioned with either saline or morphine during the animals’ dark cycle (our experimental paradigm required the separation of saline and morphine conditioning sessions by 6 h with morphine following saline to allow time for morphine to be excreted before the next training day). Our results show that nocturnal mice display typical variations in locomotor activity (Schwartz and Zimmerman, 1990) as saline-treated, control mice showed significant increases in activity counts during pairings that occurred during the dark cycle (ZT13 or ZT21) compared to pairings that occurred during the light cycle (ZT8) on the last day of conditioning (Bonferroni *post hoc* test, ZT8 vs ZT13: *p* < 0.0001; ZT8 vs ZT21: *p* < 0.0001; Fig. 5*C*). Additionally, the timing of morphine injections did influence activity counts as a pairwise comparison revealed a significant increase in activity observed when morphine was administered at ZT8 versus ZT13 on the last day of conditioning (Bonferroni *post hoc* test, conditioning day 5 (C5): *p* = 0.02; Fig. 5*D*). By conditioning day 5, differences in activity counts were not observed between saline-conditioned or morphine-conditioned animals when pairings occurred at ZT21 (Bonferroni *post hoc* test, *p* = 0.47; Fig. 5*E*,*F*), which suggests at this time point that locomotor activity reached maximal levels. Finally, our results show that our morphine training paradigm elicits robust CPP at each conditioning time point assessed (Bonferroni *post hoc* test, ZT8: *p* = 0.0002; ZT13: *p* = 0.049; ZT21: *p* = 0.032; Fig. 5*G*). These results suggest that although repeated morphine injections elicit differential changes in PVT neuronal activity, which are dependent on the timing of morphine administration, these temporal effects are not sufficient to prevent morphine-induced CPP.

## Discussion

Our results show that repeated morphine administration during the light cycle is sufficient to increase spontaneous firing of PVT neurons along with increasing PVT neuronal intrinsic membrane excitability and excitatory synaptic glutamatergic transmission at the 24-h abstinent time point. Furthermore, we found that this morphine-induced effect was absent when morphine administration or recordings occurred during the animals’ active state (i.e., dark cycle). Finally, we found that while the timing of morphine administration differentially alters PVT neuronal excitability, it did not impact morphine-induced drug-seeking behaviors measured using CPP.

### Diurnal variations of PVT firing activity and postsynaptic response to excitatory glutamatergic transmission

The diurnal variations in PVT firing observed (Fig. 1) coincided with a previous report showing that PVT neurons express high levels of spontaneous activity, both as tonic and burst firing, during darkness corresponding to the nocturnal animals’ period of activity (Kolaj et al., 2012). These variations in basal firing are correlated with alterations in intrinsic neuronal properties including an elevated membrane resistance and a lower membrane conductance (Kolaj et al., 2012). In agreement with this, we found that PVT neurons recorded from saline-treated mice during the dark cycle expressed depolarized membrane potentials and displayed increases in membrane resistance (membrane resistance: saline-treated light cycle: 562.4 ± 54.2 MΩ; saline-treated dark cycle: 1149 ± 129 MΩ; *t*_(43)_ = 4.26, *p* = 0.0001, Student’s *t*-test; resting membrane potential: saline-treated light cycle: –59.36 ± 0.90 mV; saline-treated dark cycle: –51.38 ± 1.04 mV; *t*_(64)_ = 5.810, *p* < 0.0001, Student’s *t*-test; Table 2). Furthermore, previous reports have shown that during the day, when animals are at rest, PVT neurons are “silent,” as spontaneous activity is significantly reduced (Kolaj et al., 2012). These firing properties are typical of thalamic neurons, which can express either tonic or burst firing (Jahnsen and Llinás, 1984; Crunelli et al., 2005; Steriade, 2005; Wong et al., 2013), corresponding to states of arousal; burst firing is observed during slow-wave sleep and wakefulness, whereas tonic firing is observed during wakefulness or is sufficient to induce wakefulness (McCormick and Bal, 1997; Reinagel et al., 1999; Fanselow et al., 2001; Llinás and Steriade, 2006; Ren et al., 2018). However, a unique feature of the PVT is that the neuronal firing properties are dynamically controlled by the circadian time of day (Colavito et al., 2015). Our results reiterate this circadian dependent PVT activity as, under control conditions, PVT neuronal firing in response to current injections was significantly increased during the night versus during the day (Figs. 2, 3).

One potential factor contributing to PVT diurnal variations is excitatory glutamatergic input, as PVT neurons receive excitatory synaptic input that determines their spontaneous firing discharge (Hermes and Renaud, 2011). We found that under control conditions, excitatory glutamatergic transmission at PVT synaptic connections does express diurnal variations as AMPAR/NMDAR ratios were significantly different when comparisons were made between saline-treated animals during the day versus saline-treated animals during the night (AMPAR/NMDAR ratios, saline-light cycle vs saline-dark cycle *t*_(18)_ = 2.76, *p* = 0.01, Student’s *t*-test). These results demonstrate a circadian-cycle-dependent effect on the postsynaptic response to glutamate. Additionally, although not analyzed here, presynaptic factors may also be altered by circadian cycles, including probability of glutamate release on PVT neurons, which, according to quantal theory, could impact the frequency of glutamate transmission (Redman, 1990).

We should note that some differences exist, despite some similarities of our results to other studies. We observed only two firing phenotypes from PVT neurons during dark cycle recordings in our saline-treated mice, including delayed firing and tonic firing. Others have identified three extra firing phenotypes including reluctant firing, initial burst, and single spiking (Yeoh et al., 2014). The reasons for this discrepancy are unclear, however, between the studies, a number of differences exist, including bath temperature used during recordings (22–24°C vs 33°C) and/or the PVT region recorded (mPVT vs aPVT). Additionally, other studies have shown far less tonic firing neurons during the dark phase compared to what we observed (Kolaj et al., 2012). A potential explanation for this are differences in species (mice vs rats) or treatments (saline-treated vs naive) between the studies (We understand that saline treatment is unlikely to elicit changes in PVT firing, however, repeated experimenter-mediated injections may alter levels of arousal and could potentially be responsible for the differences observed). All in all, these findings demonstrate that, due to variations that may take place from study to study, it is important to acquire the necessary number of control conditions when performing PVT neuronal recordings.

Lastly, we acknowledge that we randomly sampled PVT neurons. It is known that the PVT consists primarily of glutamatergic neurons (Christie et al., 1987; Frassoni et al., 1997; Csáki et al., 2000; Myers et al., 2014), but other populations have been identified including enkephalin, substance P, neurotensin, and galanin (Skofitsch and Jacobowitz, 1985; Melander et al., 1986; Arluison et al., 1994). Based on this, it would be useful for future studies to investigate cell-type specific activity during stages of vigilance and the corresponding effects produced by drugs of abuse.

### PVT activity following morphine administration

Twenty-four hours following a 5 d, once daily, repeated morphine administration paradigm, we found that PVT neuronal spontaneous firing (Fig. 1), intrinsic membrane excitability (Fig. 2), and response to excitatory glutamatergic transmission (Fig. 4) are all preferentially increased during light cycle morphine administration and electrophysiological recordings. Furthermore, we found that the intrinsic membrane excitability was significantly increased in tonically firing neurons originating from morphine-treated animals, but only at the maximum current injection tested (100 pA; Fig. 2*D*). This suggests that, following morphine treatment, the signal transmission on PVT neurons may be maintained over a greater range of frequencies and/or allow PVT neurons to sample greater sets of afferent signals made up of higher frequency compositions. Lastly, we did not observe any changes in PVT activity or response to excitatory synaptic inputs during dark cycle morphine administration and recordings due to the already elevated levels under control conditions.

Although the mechanisms mediating this diurnal morphine-induced shift to tonic firing are unknown, there is evidence that ion channels and/or specific neurotransmitters may mediate the observed changes. It has been reported previously that under basal conditions, T-type low-voltage-activated Ca^2+^ currents as well as hyperpolarization-activated cation currents (I_H_) are increased on PVT neurons during the dark cycle, potentially contributing to the observed increases in firing (Kolaj et al., 2012). Our results suggest that differences existed in PVT neuron I_H_ currents between saline-treated animals sampled from the light versus dark cycles measured by the voltage sag induced by hyperpolarizing current injections (*t*_(29)_ = 2.05, *p* = 0.0499, Student’s *t*-test; Table 2). However, we did not observe any changes in putative I_H_ currents when comparisons were made between saline and morphine groups during the light (*t*_(28)_ = 0.663, *p* = 0.5128, Student’s *t*-test) or dark phases (*t*_(33)_ = 0.204, *p* = 0.8397, Student’s *t*-test; see Table 2), which suggests that the morphine-induced shift may not be mediated by I_H_ currents.

Another potential mechanism mediating the morphine-induced shift to tonic firing are N-type Ca^2+^ channels and small-conductance Ca^2+^-dependent K^+^ (SK) channels, which are involved in maintaining tonic firing patterns following current injections (Wong et al., 2013). In morphine-treated animals, we observed that the frequency of tonically firing PVT neurons was not affected by the time of day that morphine was administered (two-way ANOVA with Bonferroni *post hoc* test, *p* > 0.99; Fig. 3*D*). Therefore, it is plausible that morphine administration during the light cycle may influence PVT neuronal firing in much the same way as the circadian cycle, which increases T-type and/or N-type Ca^2+^ currents along with SK-mediated currents to elicit increases in the expression of tonically firing PVT neurons.

In addition to ion channels, morphine may impact PVT neuronal firing through its modulation of neurotransmitter release. Both vasopressin and orexin, neurotransmitters whose expression are likely modulated by morphine (Aziz et al., 1981; Harris et al., 2005), elicit a switch between bursting to tonically firing phenotypes in PVT neurons (Zhang et al., 2006; Kolaj et al., 2007).

Future studies investigating the mechanisms mediating morphine’s effects on PVT neurons during inactive states will enable direct PVT neuronal manipulations to attenuate or augment morphine-induced changes. These direct manipulations have the potential to reveal whether the PVT responses to morphine during short-term abstinence are sufficient to block drug-seeking behaviors induced by stress, cues/context, or drug-priming injections. Here, we used the temporal effects observed to investigate whether context-induced morphine-seeking behaviors could be prevented by timing injections of morphine with suboptimal changes in PVT activity (ZT13) as observed from our electrophysiological experiments (Figs. 1, 4). We found that morphine-induced increases in locomotor activity were influenced by time of day, which was due to an increase in basal locomotor activity, as saline-treated animals demonstrated comparable activity counts versus morphine-treated animals. Additionally, we found that morphine conditioning at ZT8 facilitated greater activity by the fifth conditioning day compared to morphine administration at ZT13. We did not directly investigate the mechanisms and/or brain regions mediating the observed temporal effect on morphine-induced locomotor activity. However, dopamine may play a role, as it has been shown previously that dopamine expression is dependent on the circadian cycle (Schade et al., 1995; Hood et al., 2010), and that dopamine can directly influence locomotor activity (Pijnenburg et al., 1976; Isaacson et al., 1978; Fifel and Cooper, 2014).

Although timing morphine conditioning with periods of vigilance did not alter morphine-induced drug seeking as measured using the CPP paradigm (Fig. 5*F*), our electrophysiological findings, and previously published studies from others, suggest that morphine-induced PVT alterations may impact reward-seeking behaviors (Matzeu et al., 2014, 2016; Matzeu and Martin-Fardon, 2018). For example, previous studies have shown that increases in PVT glutamatergic transmission in the nucleus accumbens induces aversion and blocks seeking for natural rewards (Zhu et al., 2016; Do-Monte et al., 2017), while decreasing PVT glutamatergic transmission in the accumbens increases seeking to natural rewards and increases food consumption (Do-Monte et al., 2017; Reed et al., 2018). Furthermore, the acquisition of natural-or drug-seeking behaviors is increased (in respect to sucrose) or decreased (in respect to cocaine) with increases or decreases in PVT to nucleus accumbens transmission, respectively (Labouèbe et al., 2016; Neumann et al., 2016). In addition, it has been shown that cues predicting reward elicit decreases in PVT activity likely mediated by decreases in prefrontal cortical glutamate transmission (Otis et al., 2019). Lastly, reducing glutamatergic transmission in the PVT attenuates drug-induced reinstatement to cocaine (James et al., 2010; Yeoh et al., 2014) and inhibition of the PVT, via intraposterior PVT administration of the GABA_A_ and GABA_B_ receptor agonists muscimol and baclofen, blocked cue-induced reinstatement to cocaine (Matzeu et al., 2015). All in all, it is clear that PVT activity plays a role in reward seeking and responds to cue-reward associations suggesting that other models of substance use disorder, including self-administration, may be worthwhile models to test the timing of morphine administration on drug-seeking behaviors.

Lastly, our studies have focused on a morphine dose (10 mg/kg, i.p.) that does not produce noticeable signs of opioid withdrawal (Robinson and Kolb, 1999), but rather elicits locomotor sensitization and CPP (Spanagel et al., 1998; Mueller et al., 2002; Graziane et al., 2016). We have not tested whether escalating doses of morphine, that are known to produce somatic signs of withdrawal, would elicit similar effects on PVT neuronal activity in either the light or dark cycles. Evidence suggests that independent neurocircuits may be implicated in the somatic and affective responses to acute opioid withdrawal (Aston-Jones et al., 1999; Delfs et al., 2000). Therefore, understanding how PVT neurons are affected by varying the morphine dose may have implications in different states of opioid abstinence, including a state of early abstinence in opioid-dependent patients known to experience withdrawal symptoms or a state of prolonged abstinence in patients having already undergone opioid withdrawal.

### Sleep disturbances and relapse

Our results indicate that during morphine abstinence, PVT neuronal firing and excitability is preferentially increased during the animals’ inactive state (Figs. 1-Figs. 3), suggesting that the basal rhythms of PVT activity are disrupted following repeated morphine exposure. The PVT is directly involved in mediating wakefulness via increases in tonic firing (7–10 Hz; Ren et al., 2018). Therefore, it is plausible, given that the PVT regulates light-induced phase shifts in the circadian cycle (Salazar-Juárez et al., 2002), that during short-term morphine abstinence, increases in PVT activity during inactive states may induce sleep disturbances via facilitating wakefulness despite external stimuli signaling a period of dormancy. Future studies are required to directly test whether the PVT plays a role in the already observed morphine-induced circadian rhythm shifts, which induce a phase delay or phase advance in circadian oscillations depending on the time of opioid administration (Marchant and Mistlberger, 1995). Doing so may reveal a key brain region mediating morphine-induced changes in circadian rhythms.

Lastly, there is growing evidence that sleep difficulties are a potential risk factor for opioid use disorder and relapse (Oyefeso et al., 1997; Hasler et al., 2012; Logan et al., 2014, 2018). In rats, morphine delays the onset of REM sleep and in humans, morphine decreases the number and duration of REM periods, delays REM onset, increases the waking state during the early night, and increases NREM light sleep (stages 1 and 2), while decreasing NREM deep sleep (stages 3 and 4; Kay et al., 1969; Arankowsky-Sandoval and Gold, 1995). These disruptions in sleep patterns are correlated with drug-seeking behaviors as an interaction between time of day, and drug-seeking behaviors have been shown in rats, who will self-administer more heroin and exhibit more drug-seeking behaviors when training takes place during the dark phase versus the light phase (Coffey et al., 2018). Additionally, light/dark cycle alterations have been shown to alter morphine-induced CPP (Tahsili-Fahadan et al., 2005). Taken together, these results suggest that drug-seeking behaviors may be avoidable in some patients if corrections can be made to drug-induced sleep disturbances. Despite our results showing that context-induced morphine-seeking behaviors were not dependent on time of morphine injection, it does not rule out that a chronotherapy approach with morphine may alleviate drug-seeking behaviors, which may be investigated in future studies using other models of substance use disorder including self-administration. The idea that chronotherapy may mitigate rewarding properties of drugs is in line with evidence suggesting that chronopharmacology impacts other morphine-induced effects, including analgesia (Yoshida et al., 2003; Yu et al., 2015).

## Synthesis

Reviewing Editor: Karen Szumlinski, University of California at Santa Barbara Decisions are customarily a result of the Reviewing Editor and the peer reviewers coming together and discussing their recommendations until a consensus is reached. When revisions are invited, a fact-based synthesis statement explaining their decision and outlining what is needed to prepare a revision will be listed below. The following reviewer(s) agreed to reveal their identity: NONE.

Following consultation with two experts reviewers, we have come to the decision of Revise-Re-Review based on some concerns that were raised. Overall, we all considered the report to be well-written, the data new and interesting and of relevance to circadian disruption that often accompanies addiction. The report should be of interest not only to those studying the neurobiology of addiction, but also those studying circadian rhythms and sleep disorders. While the report received support from both reviewers, some major concerns were raised. A major concern raised by Reviewer 1 relates to the relatively large breadth of neuronal sampling within the PVT. There is no consideration or description of subregional differences in behavioral or electrophysiological measures along the anterior-posterior axis of the PVT and such information should be included in the revision. As the authors likely have information pertaining to the localization of their electrodes, we feel that they can address issues related to the neuroanatomical specific of observed effects rather readily (i.e., within a 2-month period). Reviewer 2 expressed concerns regarding the dose-range of morphine employed in the study, suggesting, in the very least, that the authors consider in their Discussion how the data might play out if higher morphine doses were tested. In addition to these major concerns, both reviewers expressed other concerns (the majority of which are relatively minor) that should be addressed in the revised version of this report. Please find these specific comments below:

Reviewer 1

This study examines the effects of repeated morphine exposure on firing and intrinsic properties in PVT neurons. The authors generated acute PVT slices from mice that had received morphine during their light or dark periods and assessed the impact on physiology using whole-cell techniques. Additionally, they evaluated morphine conditioned place preference (CPP) after morphine pairings that occurred during the light or dark cycle. The authors provide a compelling case that light-period morphine exposure elevates spiking and excitability in the PVT, bringing the properties of these cells in line with what is observed during the animal's dark period (awake/aroused state). Due to the interconnectivity of the PVT with regions that govern circadian rhythms, assessing changes in spike rate after drug exposure is behaviorally relevant and could have important clinical implications. This case is strengthened by the fact that the authors use multiple electrophysiological methods to assess this and find several differences between light- and dark-period morphine administration. While it is disappointing to not see any differences in CPP, I do not think that this detracts from the value of knowing there are physiological differences between the effects of light- and dark-period drug administration. The manuscript is well-written and concise, and the findings would be of interest to neuroscientists who study the effects of drug exposure as well as those that study circadian rhythms. However, I think that some key issues need to be addressed to make the story solid and informative, as outlined below.

Major Points

1) The PVT spans a large anatomical range, and it seems like the authors sampled neurons throughout the anterior-posterior range during recordings. Anterior and posterior PVT have been shown to have numerous functional and anatomical (inputs/outputs, particularly with regard to SCN connectivity - see Alamilla et al., 2015, EJN), so I do not think it's acceptable to use such a large range. It would be important to note whether any subregion-level differences are seen in the data. For example, does the presence/magnitude of morphine-evoked activity elevation differ across the PVT? What about the circadian changes in general? This could provide some insight into what the downstream effects of elevated activity might be, and/or what inputs could contribute to the changes. If the authors do not have the information to do this with existing data or don't have a large enough dataset to do so, it would be necessary to collect a dataset that directly compares the anterior and posterior subregions, at least with regard to light vs dark timepoints. Alternatively, since the Kolaj et al. paper that the authors cite for circadian changes in the PVT specifically focuses on the aPVT, it could be a better idea to only include electrophysiological data from this region (which may entail collecting more data to replace the more posterior cells). One important methodological issue here is that there may be different populations of PVT neurons with varying intrinsic properties (or AMPAR/NMDAR ratios) across the anterior-posterior spread. If cells aren't being similarly sampled in the saline-treated and morphine-treated animals, then it would be impossible to compare any changes in firing rate or intrinsic properties. Additionally, while coordinates certainly vary from lab to lab, I'm a bit concerned by the range of “-0.94 to -2.18” (line 112), given that many groups would define anterior PVT as starting somewhere around -0.3 and would likely put the caudal limit around -0.94 (Yeoh et al., 2014, for example), which wouldn't even overlap with the authors' region. This makes it very hard to put this paper in the context of what is already known about the PVT and drug-evoked changes there.

2) While the authors note some of these values in the discussion (343-346), they should additionally include a table or graph of all intrinsic properties collected in the four groups (Light-saline, Light-morphine, Dark-saline, Dark-morphine), with appropriate statistical comparisons. It'd be helpful to see the resting membrane potential, membrane resistance, and rheobase in particular, but the authors should also analyze their data for the presence of IH currents as well, since they note in the discussion that alterations of such currents could contribute to the increased excitability (and there are diurnal fluctuations in the Kolaj et al. paper). It should be discernible in the traces that include hyperpolarizing current injections and can be analyzed by evaluating the voltage sag. The authors should already have all of this information, based off of the recordings they've described in the paper, so it should be straightforward to organize the data into a table. One of the cited papers (Yeoh et al., 2014) provides a nice example of how to do this.

Minor Points

1) The introduction briefly notes that the authors use CPP rather than operant self-administration to isolate mechanisms mediating contextual associations, but that does not seem to be a major interest of the study, judging by the rest of the introduction and the discussion. There should either be a bit more justification for why this behavioral paradigm was chosen, and/or why the PVT was the region of interest.

2) In the Results section (238-243) and legend for Figure 2D, the authors note that the spike number analysis for tonically spiking neurons is not statistically significant. However, there do appear to be emerging differences at the higher current injections, as evidenced by the post-hoc testing and visual inspection of the graph. This could mean that tonically spiking neurons are significantly affected by morphine treatment, but that the effect is to enhance their firing rate with larger current injections - potentially making their maximum firing rate significantly different. If they authors do collect any additional data to address the points above, it would be a good idea to do a couple of larger current injections to determine if this is the case. At the very least, this point should be addressed in the text.

3) The “Repeated systemic injections of saline or morphine” methods section appears to be incomplete - the end of this section is in fragments (lines 94-97), making it hard to discern the rationale for this particular drug treatment paradigm. The authors should clarify this section.

4) Likely just a typographical error, but the “Electrophysiology” section (line 132) should be corrected to clarify the range of injected currents (presumably it's -100 to +100 pA).

5) In “Acute Brain Slice Preparation” (line 104-105), it's a bit unclear to me what “lateral hemispheres were removed” means in this context. It'd be helpful to note how much of the slice is being cut away during this step.

6) Authors should provide slightly more detail regarding where the stimulating electrode is placed for the AMPAR/NMDAR experiments, and how far the electrode typically is from the recorded cells.

7) 340: It's unclear what is meant by “depolarized membrane resistance” - this should likely be either “depolarized membrane potential” and/or “elevated membrane resistance.” Similarly, they then go on to state values for membrane resistance in mV, which should certainly be in MOhms.

8) I found the timing of various experiments and drug treatments a bit hard to discern throughout the paper. In particular, the timing of the final CPP experiments and how that relates to the timing of the earlier electrophysiological recordings is somewhat complicated to figure out, and might be better served by a high-level diagram of some sort (including the repeated morphine treatments and euthanasia/slice times for experiments in Figures 1-4). Be sure to note which groups of mice are on a reverse light/dark cycle.

9) If there are any other papers that examine spiking phenotypes and intrinsic properties in the PVT, the authors should include a section in the discussion that compares the 4 categories they observed to what has already been demonstrated. For example, is it typical to see nearly all PVT neurons exhibit tonic firing during the dark cycle?

10) The authors should note that the experiments are being done with acute slices from mice in the abstract. In its current state, I would assume the authors had conducted in vivo recordings based off of the abstract.

Reviewer 2

Overall this is a well performed study. They combine electrophysiological recordings together with injections of morphine to determine whether the time of day of drug administration affects PVT neuron activity.

While the role of PVT in arousal and drug motivated behaviour is well described and referenced - the reason for altering the timing of drug administration seems less clear. Is this because opioid users tend to administer drug at night or is there some other clinical reason for this questions that I am missing?

The authors used a dose of morphine at the lower end of the range that doesn't produce frank withdrawal. I am wondering whether its important to determine whether a higher dose that produces withdrawal might be necessary to substantiate the claim that morphone during the active period does not affect PVT excitability during the dark cycle - or CPP?

Given that no assessment of sleep was made - the authors spend too much time discussing their data in this context and this should be reduced.

## Author Response

Dear Dr. Szumlinski,

We are extremely pleased that our manuscript is potentially suitable for eNeuro. Additionally, we are very grateful for the reviewers' insightful comments and suggestions. By addressing each reviewer's concerns, we feel that our manuscript has been significantly strengthened. Before we address each concern raised (below), we would like to point out a couple of changes that we have made in the manuscript. First, after discussions with a statistician, we are now performing two-way repeated measures ANOVA for Figs. 2C, D, 3C, 3D, 5C, and 5D. Repeated measures was used to analyze changes in the mean over varying current injections (Figs. 2 and 3) or days (Fig. 5). The two-way repeated measures ANOVA will replace the two-way ordinary ANOVA used in our initial submission. This correction did not alter our pairwise comparisons as shown in our initial submission and therefore, all of post-hoc analysis is consistent between this revised manuscript and our initial submission. Additionally, we included a justification for our statistical approaches, which we feel provides rationale for our statistical analyses (lines 189-197). Second, to avoid being redundant, we removed mean{plus minus}SEM from the text and only included it in the figures. This decision was based on reviewer comments from another manuscript of ours that is currently under review, but if the reviewers for this manuscript disagree, we are open to suggestions.

Reviewer #1:

Major points:

1) The PVT spans a large anatomical range, and it seems like the authors sampled neurons throughout the anterior-posterior range during recordings...

We want to thank the reviewer for bringing up this concern as this is an important correction that requires clarification. All recordings were made of a region spanning between Bregma -0.94 mm and -2.18 mm, which corresponds to the middle PVT (mPVT) and excludes the anterior PVT (aPVT) and posterior PVT (pPVT). We are extremely grateful that the reviewer pointed out that we inadvertently inaccurately stated that this region covers aPVT and pPVT and have made the necessary correction to the Methods Section (lines 112-116).

We specifically targeted the middle PVT because this region is implicated in wakefulness (Ren et al., 2018). Doing so, we feel, aligns with the studies intended purpose, which is to investigate whether timing morphine injections with specific states of arousal can influence drug-context associations.

We agree with the reviewer that comparisons between aPVT, mPVT, and pPVT would be extremely valuable and is a comparison that we are planning to target in our continuing studies.

2) a. While the authors note some of these values in the discussion (343-346), they should additionally include a table or graph of all intrinsic properties collected in the four groups (Light-saline, Light-morphine, Dark-saline, Dark-morphine), with appropriate statistical comparisons.

b. It'd be helpful to see the resting membrane potential, membrane resistance, and rheobase in particular, but the authors should also analyze their data for the presence of IH currents as well, since they note in the discussion that alterations of such currents could contribute to the increased excitability (and there are diurnal fluctuations in the Kolaj et al. paper).

We have included this information in Table II and a discussion regarding the IH currents has been added (lines 421-428). The reviewer may notice that the resting membrane potentials (RMP) have a larger n. This is because we have included the RMP of the neurons recorded in Figure 1.

Minor Points

1) The introduction briefly notes that the authors use CPP rather than operant self-administration to isolate mechanisms mediating contextual associations, but that does not seem to be a major interest of the study, judging by the rest of the introduction and the discussion. There should either be a bit more justification for why this behavioral paradigm was chosen, and/or why the PVT was the region of interest.

This has been addressed in the introduction as we aligned the use of CPP with this manuscript's focus on morphine's effects on brain regions implicated in wakefulness and the potential link to controlling addiction-like behaviors (lines 61-66). We tried to keep this concise, however, if the reviewer thinks that further clarification is required, we are happy to do so.

2) In the Results section (238-243) and legend for Figure 2D, the authors note that the spike number analysis for tonically spiking neurons is not statistically significant. However, there do appear to be emerging differences at the higher current injections, as evidenced by the post-hoc testing and visual inspection of the graph. This could mean that tonically spiking neurons are significantly affected by morphine treatment, but that the effect is to enhance their firing rate with larger current injections - potentially making their maximum firing rate significantly different. If they authors do collect any additional data to address the points above, it would be a good idea to do a couple of larger current injections to determine if this is the case. At the very least, this point should be addressed in the text.

We have now addressed this in the text (lines 248-253 and lines 421-428).

3) The “Repeated systemic injections of saline or morphine” methods section appears to be incomplete - the end of this section is in fragments (lines 94-97), making it hard to discern the rationale for this particular drug treatment paradigm. The authors should clarify this section.

We have made the necessary clarifications (lines 93-96).

4) Likely just a typographical error, but the “Electrophysiology” section (line 132) should be corrected to clarify the range of injected currents (presumably it's -100 to +100 pA).

We have adjusted this to accurately read -100 to +100 pA.

5) In “Acute Brain Slice Preparation” (line 104-105), it's a bit unclear to me what “lateral hemispheres were removed” means in this context. It'd be helpful to note how much of the slice is being cut away during this step.

We have clarified this in the text (lines 103-105).

6) Authors should provide slightly more detail regarding where the stimulating electrode is placed for the AMPAR/NMDAR experiments, and how far the electrode typically is from the recorded cells.

We have updated this in the Methods Section (lines 142-143).

7) 340: It's unclear what is meant by “depolarized membrane resistance” - this should likely be either “depolarized membrane potential” and/or “elevated membrane resistance.” Similarly, they then go on to state values for membrane resistance in mV, which should certainly be in MOhms.

We have clarified this point and corrected our units as the reviewer suggested.

8) I found the timing of various experiments and drug treatments a bit hard to discern throughout the paper. In particular, the timing of the final CPP experiments and how that relates to the timing of the earlier electrophysiological recordings is somewhat complicated to figure out, and might be better served by a high-level diagram of some sort (including the repeated morphine treatments and euthanasia/slice times for experiments in Figures 1-4). Be sure to note which groups of mice are on a reverse light/dark cycle.

This is a valid point and something that we were concerned about as well. A diagram is now included in the first and last figure. Additionally, we changed our time points to Zeitgeber time for clarity.

9) If there are any other papers that examine spiking phenotypes and intrinsic properties in the PVT, the authors should include a section in the discussion that compares the 4 categories they observed to what has already been demonstrated. For example, is it typical to see nearly all PVT neurons exhibit tonic firing during the dark cycle?

This important point has been included in the discussion (lines 381-495).

10) The authors should note that the experiments are being done with acute slices from mice in the abstract. In its current state, I would assume the authors had conducted in vivo recordings based off of the abstract.

Yes, this is critical. We have clarified this in the abstract.

Reviewer 2

1) While the role of PVT in arousal and drug motivated behaviour is well described and referenced - the reason for altering the timing of drug administration seems less clear. Is this because opioid users tend to administer drug at night or is there some other clinical reason for this questions that I am missing?

This is a valid concern that we have attempted to clarify in the text (lines 61-66 and 522-524). In summary, chronopharmacology has been shown to impact morphine-induced analgesia (Yoshida et al., 2003; Yu et al., 2015; cited in the manuscript). Given that the conditioned place preference (CPP) paradigm and PVT activity are both influenced by circadian rhythms (Abarca et al., 2002; Kurtuncu et al., 2004; Peng et al., 1995; Novak and Nunez, 1998; Kolaj et al., 2012-cited in the manuscript) and that PVT activation impacts cocaine-induced conditioned place preference (Browning et al, 2014-cited in the manuscript), we attempted to time the morphine dose with different PVT activation states to determine whether morphine-induced CPP was influenced by chronopharmacological effects. If so, we intended to extrapolate our findings to the patient population to investigate whether timing opioid administration with specific times coinciding with a patient's circadian cycle may reduce the morphine-induced circadian shift and thereby decrease the desire to continue to take opioids.

2) The authors used a dose of morphine at the lower end of the range that doesn't produce frank withdrawal. I am wondering whether it's important to determine whether a higher dose that produces withdrawal might be necessary to substantiate the claim that morphine during the active period does not affect PVT excitability during the dark cycle - or CPP?

This is an excellent question and something that we have considered, but did not include in our investigation to avoid confounding factors elicited by somatic symptoms of withdrawal and to keep consistent with previous investigations demonstrating the range of morphine that elicits CPP (see Tabaeizadeh M, http://dx.doi.org/10.1016/j.bbr.2012.09.010). However, given the importance of this point, we now have included a section in the Discussion addressing this (lines 481-491) and we strongly feel that this is an excellent future investigation that is worth studying.

3) Given that no assessment of sleep was made - the authors spend too much time discussing their data in this context and this should be reduced.

We understand the reviewer's concerns given that we did not assess sleep. We included this in the discussion because of our strong belief that chronotherapy approaches for opioids may be an avenue worth studying with our aim to encourage the field to at the very least consider chronopharmacology as a potential influencing factor in substance use disorders. Given that the region of the PVT investigated here is implicated in arousal states (Ren et al., 2018), the link between sleep disturbance and opioid use may be explained by morphine's effects on specific brain regions. Including this discussion point may drive studies to investigate opioid-induced alterations in brain regions implicated in circadian rhythms, potentially adding valuable information regarding the mechanisms mediating opioid-induced sleep disturbances. Therefore, we would like to keep this discussion point at its current length.
